# Urinary activated leukocyte cell adhesion molecule as a novel biomarker of lupus nephritis histology

**DOI:** 10.1186/s13075-020-02209-9

**Published:** 2020-05-27

**Authors:** Huihua Ding, Cheng Lin, Jingyi Cai, Qiang Guo, Min Dai, Chandra Mohan, Nan Shen

**Affiliations:** 1 Department of Rheumatology, Shanghai Institute of Rheumatology, Renji Hospital, Shanghai Jiao Tong University School of Medicine, 145 Shandong (M) Rd, Shanghai, 200001 China; 2 Department of Biomedical Engineering, University of Houston, 3517 Cullen Blvd, Room 2027, Houston, TX 77204-5060 USA; 3 China-Australia Centre for Personalized Immunology, Renji Hospital, School of Medicine, Shanghai Jiao Tong University, Shanghai, China; 4 State Key Laboratory of Oncogenes and Related Genes, Shanghai Cancer Institute, Renji Hospital, Shanghai Jiao Tong University School of Medicine (SJTUSM), Shanghai, 200032 China; 5 Shenzhen Futian Hospital for Rheumatic Diseases, Shenzhen, 518040 China; 6 Center for Autoimmune Genomics and Etiology, Cincinnati Children’s Hospital Medical Center, Cincinnati, OH USA; 7 Department of Pediatrics, University of Cincinnati College of Medicine, Cincinnati, OH USA

**Keywords:** Lupus nephritis, Urinary biomarker, ALCAM, Renal histopathology, Activity index, Chronicity index

## Abstract

**Background:**

Lupus nephritis (LN) is one of the most severe complications of SLE patients. We aim to validate urinary ALCAM as a biomarker in predicting renal disease histpathology in a Chinese lupus cohort.

**Methods:**

In this cross-sectional study, a total of 256 patients and controls were recruited. Urinary levels of ALCAM were determined by ELISA. Renal histopathology was reviewed by an experienced renal pathologist.

**Results:**

Urinary ALCAM levels were significantly increased in active LN patients when compared to active SLE patients without renal involvement (*p* < 0.001), inactive LN patients (*p* = 0.023), inactive SLE patients without renal involvement (*p* < 0.001), and healthy controls (*p* < 0.001). Correlation analysis revealed a positive correlation between urinary ALCAM and general disease activity—SLEDAI score (*r* = 0.487, *p* < 0.001), as well as renal disease activity—rSLEDAI (*r* = 0.552, *p* < 0.001) and SLICC RAS (*r* = 0.584, *p* < 0.001). Urinary ALCAM also correlated with lab parameters including 24-h urine protein, hemoglobin, and complement 3. Moreover, urinary ALCAM levels were significantly increased in class III and IV (proliferative) LN as compared to those in class V (membranous) LN. It outperformed conventional biomarkers (anti-dsDNA antibody, C3, C4, proteinuria) in discriminating the two groups of LN. On renal histopathology, urinary ALCAM levels correlated positively with activity index (*r* = 0.405, *p* < 0.001) but not chronicity index (*r* = 0.079, *p* = 0.448).

**Conclusion:**

Urinary ALCAM is a potential biomarker for predicting renal pathology activity in LN and may serve as a valuable surrogate marker of renal histopathology.

## Introduction

Systemic lupus erythematosus (SLE) is an autoimmune disease that has a wide impact on multiple organs, including the kidneys. Approximately 40–70% of SLE patients develop lupus nephritis (LN) during their disease courses [1]. LN is one of the most common manifestations of SLE and an important driver of mortality and morbidity in SLE [2]. Ten to 30% of patients with severe LN progress to end-stage renal disease within 15 years despite aggressive immunosuppressive therapy [3]. Currently, renal biopsy remains the gold standard for the diagnosis and management of LN. Although it is essential for adequate diagnosis, determining a treatment regimen, and predicting prognosis in LN patients, the invasive nature and associated risks have limited its use, especially during follow-up. Conventional biomarkers, such as anti-double-stranded DNA (dsDNA) antibodies and complement levels, lack sensitivity and specificity in the diagnosis of LN or in the assessment of renal pathology [4, 5]. Thus, there is an unmet need for non-invasive biomarkers to assess renal histology, predict renal prognosis, and ultimately guide the treatment of LN.

High-throughput proteomics-based approaches have provided an efficient way for screening potential biomarkers worthy of further validation. Our preliminary aptamer-based proteomic screening study identified several novel urinary biomarkers for active LN [6]. Among these proteins, activated leukocyte cell adhesion molecule (ALCAM or CD166) turned out to be a promising biomarker for LN. ALCAM is a cell surface glycoprotein which belongs to the immunoglobulin super family [7]. Soluble ALCAM in body fluids is produced through proteolytic cleavage by ADAM17 or alternative splicing [8, 9]. It plays an important role in T cell activation and immune cell adhesion and migration through its interaction with CD6 [10, 11]. A microarray study of glomeruli from MRL/lpr mice revealed increased ALCAM gene expression in the kidneys of mice with LN [12]. The role of ALCAM as a biomarker in renal disease was recently reported in type 2 diabetic nephropathy [13]. In this study, we aim to further validate the performance of urinary ALCAM as a biomarker in assessing disease activity and renal histopathology in a Chinese LN cohort.

## Methods

### Study subjects

A total of 96 biopsy-proven active LN, 59 active SLE without LN, 10 inactive LN patients, 63 inactive SLE patients without renal involvement, and 28 age- and gender-matched healthy controls were recruited. All subjects were of Chinese ethnicity. All SLE patients fulfilled the 1997 revised American College of Rheumatology (ACR) classification criteria for SLE or 2012 SLICC criteria for SLE [14]. For active LN patients, all samples were collected at the time of renal biopsy. Detailed demographic and clinical characteristics are summarized in Table 1.

**Table 1:** Demographic and clinical characteristics of study subjects

	SLE				HC
	aLN	aNR	iLN	iNR	
*N*	96	59	10	63	28
Age (years) (mean ± SD)	34.7 ± 11.7	35.8 ± 15.0	37.2 ± 17.7	43.8 ± 29.8	32.0 ± 11.6
Female, *n* (%)	91 (94.8%)	55 (93.2%)	9 (90%)	60 (95.2%)	23 (82.1%)
Disease duration (years) (IQR)	4.9 (8.1)	2.1 (11.8)	4.3 (9.7)	2.8 (9.6)	0 (0)
**Clinical characteristics,*n*(%)**					
Fever	7 (7.3%)	11 (18.6%)	0 (0%)	10 (15.8%)	N/A
Lymphadenopathy	3 (3.1%)	2 (3.4%)	0 (0%)	1 (1.6%)	N/A
Malar rash	15 (15.6%)	14 (23.7%)	2 (20%)	7 (11.1%)	N/A
Mucosal ulceration	2 (2.1%)	3 (5.1%)	0 (0%)	1 (1.6%)	N/A
Alpecia	5 (5.2%)	6 (10.2%)	0 (0%)	1 (1.6%)	N/A
Vasculitis	6 (6.3%)	5 (8.5%)	0 (0%)	1 (1.6%)	N/A
Raynoud’s phenomenon	3 (3.1%)	1 (1.7%)	1 (10%)	1 (1.6%)	N/A
NPSLE	0 (0%)	6 (10.2%)	0 (0%)	1 (1.6%)	N/A
Myositis	0 (0%)	1 (1.7%)	0 (0%)	0 (0%)	N/A
Arthralgia/arthritis	5 (5.2%)	13 (22.0%)	0 (0%)	3 (4.8%)	N/A
Endocarditis	9 (9.4%)	4 (6.8%)	2 (20%)	2 (3.2%)	N/A
PAH	3 (3.1%)	8 (13.6%)	1 (10%)	2 (3.2%)	N/A
ILD	3 (3.1%)	3 (5.1%)	1 (10%)	2 (3.2%)	N/A
Pleural effusion	4 (4.2%)	1 (1.7%)	0 (0%)	4 (6.3%)	N/A
Leukocytopenia	10 (10.4%)	17 (28.8%)	0 (0%)	6 (9.5%)	N/A
Hemolytic anemia	0 (0%)	2 (3.4%)	0 (0%)	1 (1.6%)	N/A
Thrombocytopenia	6 (6.3%)	14 (23.7%)	1 (10%)	8 (12.7%)	N/A
Gastrointestinal vasculitis	0 (0%)	0 (0%)	3 (30%)	3 (4.8%)	N/A
rSLEDAI, median (IQR)	8 (8)	0 (0)	0 (0)	0 (0)	N/A
SLEDAI, median (IQR)	12 (8)	8 (6)	4 (3)	4 (2)	N/A
**Laboratory tests, median (IQR)**					
24-h urine protein (g/24 h)	3.0 (4.4)	0.2 (0.2)	0.4 (0.7)	0.1 (0.1)	N/A
ANA positive/tested	82/82	52/52	8/8	58/58	N/A
C3 (g/L)	0.57 (0.39)	0.60 (0.37)	0.70 (0.67)	0.77 (0.47)	N/A
C4 (g/L)	0.10 (0.08)	0.08 (0.11)	0.14 (0.1)	0.14 (0.13)	N/A
dsDNA (IU/mL)	25.67 (27.10)	28.56 (26.17)	13.15 (27.85)	25.07 (45.31)	N/A
**Renal pathology (ISN/RPS classification),*n*(%)**					
Class I	0 (0%)	N/A	N/A	N/A	N/A
Class II	1 (1.0%)	N/A	N/A	N/A	N/A
Class III/III + V	20 (20.8%)	N/A	N/A	N/A	N/A
Class IV/IV + V	54 (56.3%)	N/A	N/A	N/A	N/A
Class V	21 (21.9%)	N/A	N/A	N/A	N/A
Class VI	0 (0%)	N/A	N/A	N/A	N/A
**Comorbidity,*n*(%)**					
RA	0 (0%)	0 (0%)	0 (0%)	1 (1.6%)	N/A
SS	2 (2.1%)	2 (3.4%)	1 (10%)	4 (6.3%)	N/A
APS	0 (0%)	2 (3.4%)	0 (0%)	1 (1.6%)	N/A
Osteoporosis	2 (2.1%)	2 (3.4%)	0 (0%)	2 (3.2%)	N/A
AVN of femoral head	10 (10.4%)	4 (6.8%)	0 (0%)	1 (1.6%)	N/A
Hypertension	20 (20.8%)	1 (1.7%)	1 (10%)	5 (7.9%)	N/A
Diabetes mellitus	3 (3.1%)	2 (3.4%)	0 (0%)	1 (1.6%)	N/A
Hyperlipidemia	4 (4.2%)	1 (1.7%)	0 (0%)	0 (0%)	N/A

*aLN* active LN group, *aNR* active SLE without renal involvement group, *iLN* inactive lupus nephritis group, *iNR* inactive SLE without renal involvement group, *HC* healthy control group, *NPSLE* neuropsychiatric systemic lupus erythematosus, *PAH* pulmonary arterial hypertension, *ILD* interstitial lung disease, *SLEDAI* Systemic Lupus Erythematosus Disease Activity Index, *rSLEDAI* renal SLEDAI, *ANA* anti-nuclear antibody, *C3* complement 3, *C4* complement 4, *RA* rheumatoid arthritis, *SS* Sjogren’s syndrome, *APS* antiphospholipid syndrome, *AVN* avascular necrosis

### Disease assessment

Systemic Lupus Erythematosus Disease Activity Index (SLEDAI) (version SLEDAI-2k) [15] and renal SLEDAI (refers to the total score of the four kidney-related parameters in SLEDAI) were calculated based upon the review of patients’ medical records and laboratory tests at the time of sample collection, which were used to assess disease activity in SLE patients. SLICC renal activity score (SLICC RAS) was also calculated to assess renal activity in active LN patients. SLICC RAS was calculated as follows: proteinuria 0.5–1 g/day = 3 points, proteinuria > 1–3 g/day = 5 points, proteinuria > 3 g/day = 11 points, urine red blood cell count > 5/hpf = 3 points, and urine white blood cell count > 5/hpf = 1 point [15]. Renal biopsies were reviewed and classified by an experienced renal pathologist who was blinded to the design and results of the study, using the 2004 International Society of Nephrology/Renal Pathological Society (ISN/RPS) classification [16]. Activity index and chronicity index of renal pathology were calculated as described elsewhere [16].

In the current study, SLE patients were divided into four groups (Table 1): active LN group (aLN), active SLE without renal involvement group (aNR), inactive LN patients (iLN), and inactive SLE patients without renal involvement (iNR). The active LN group of patients was renal biopsy-proven LN patients with SLEDAI ≥ 6 and rSLEDAI ≥ 4 (with biopsy-concurrent urine samples). The active SLE without renal involvement group of patients was defined as SLEDAI ≥ 6 and rSLEDAI = 0. Inactive LN patients had a history of LN with SLEDAI ≤ 4 and rSLEDAI = 0 at the time of the study. Inactive SLE patients without renal involvement had no history of renal involvement with SLEDAI ≤ 4 and rSLEDAI = 0 at the time of the study.

### Measurement of urinary ALCAM

Random urine was collected from each patient in a 50-mL sterile container. Urine samples were mixed well and aliquoted into 5-mL tubes and stored at − 80 °C until use. Urine samples were thawed and centrifuged before use. Supernatants were used for the assays. Urinary ALCAM levels were measured in urine samples by ELISA assay using human ALCAM ELISA kit (DY656) from R&D Systems (Minneapolis, MN, USA) according to the manufacturer’s instructions. All urine samples were diluted 1:50. Urinary ALCAM levels were normalized by urine creatinine levels. Urine creatinine levels were measured by Creatinine Parameter Assay Kit (KGE005, R&D Systems, Minneapolis, MN).

### Measurement of anti-dsDNA antibody and complement levels

Serum levels of anti-dsDNA antibody were measured using a Farr immunoprecipitation assay (reference range 0–7 IU/mL). Serum levels of complement component 3 (C3; reference range 0.9–1.8 g/L) and complement component 4 (C4; reference range 0.1–0.4 g/L) were measured by turbidimetric immunoassay.

### Statistical analysis

Data are expressed as mean (SD) for continuous variables with normal distribution, median (interquartile range (IQR)) for continuous variables with non-normal distribution, and counts and percentage for dichotomous variables. The Kolmogorov–Smirnov tests were used to test normality of the data. Comparison between two groups was performed using a Student’s *t* test when the data were distributed normally. Otherwise, a non-parametric Mann–Whitney *U* test was used. We used one-way analysis of variance (ANOVA) to compare three or more groups of normally distributed data, or Kruskal–Wallis *H* test for non-normally distributed data. Pearson’s method was used for correlation analysis in continuous and normally distributed data. Otherwise, the non-parametric Spearman’s method was used. We generated a receiver operating characteristic (ROC) curve to assess the performance of urinary ALCAM as a marker for lupus nephritis. Optimal cutoff value was calculated using Youden’s Index. The sensitivity, specificity, positive predictive value (PPV), and negative predictive value (NPV) were calculated using 2 × 2 contingency tables. A two-tailed *p* value < 0.05 was considered statistically significant. All statistical analyses were performed using SPSS 25 software (IBM Corp., Armonk, New York, USA), and figures were plotted using GraphPad Prism 7.0 (GraphPad Software Inc. La Jolla, CA, USA).

### Ethics and consent

Informed consent was obtained from all the participants before beginning the study. The study was approved by the ethics committee of Renji Hospital, Shanghai JiaoTong University School of Medicine, Shanghai, China.

## Results

### Elevated urinary ALCAM in active lupus nephritis patients

**Fig. 1:**
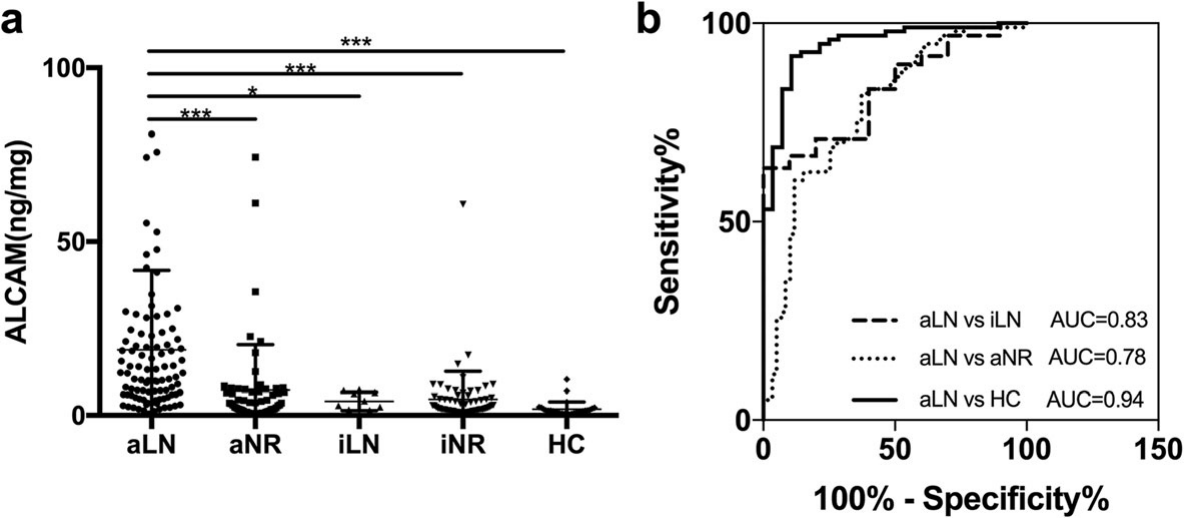
Urinary ALCAM levels were elevated in active LN patients. **a** Significant increase of urinary ALCAM in aLN patients (11.50 IQR (16.79) ng/mg) when compared to those in aNR (3.51 IQR (6.20) ng/mg), iLN (3.46 IQR (5.14) ng/mg), iNR (2.45 IQR (4.04) ng/mg), and HC (1.79 IQR (1.23) ng/mg). **b** Receiver operating characteristic curve analysis of urinary ALCAM in discriminating aLN from aNR, iLN, and HC. aLN, active LN; aNR, active SLE without renal involvement; iLN, inactive LN; iNR, inactive SLE without renal involvement; HC, healthy control; AUC, area under the curve. *p < 0.05; ***p < 0.001

In this cross-sectional study, urinary ALCAM levels were significantly increased in active LN patients (11.50 IQR (16.79) ng/mg) when compared to active SLE patients without renal involvement (3.51 IQR (6.20) ng/mg, *p* < 0.001), inactive LN patients (3.46 IQR (5.14) ng/mg, *p* = 0.023), inactive SLE patients without renal involvement (2.45 IQR (4.04) ng/mg, *p* < 0.001), and healthy controls (1.79 IQR (1.23) ng/mg, *p* < 0.001) (Fig. 1a). In order to assess the diagnostic performance of urinary ALCAM, we further performed the receiver operating curve (ROC) analysis. As indicated in Fig. 1b, the area under the curve (AUC) value was 0.94 (95% CI 0.90 to 0.99; *p* < 0.001) for active LN versus healthy control, 0.78 (95% CI 0.70 to 0.86; *p* < 0.001) for active LN versus active SLE without renal involvement, and 0.83 (95% CI 0.73 to 0.93; *p* < 0.001) for active LN versus inactive LN.

### Correlation of urinary ALCAM with disease activity and laboratory parameters

**Fig. 2:**
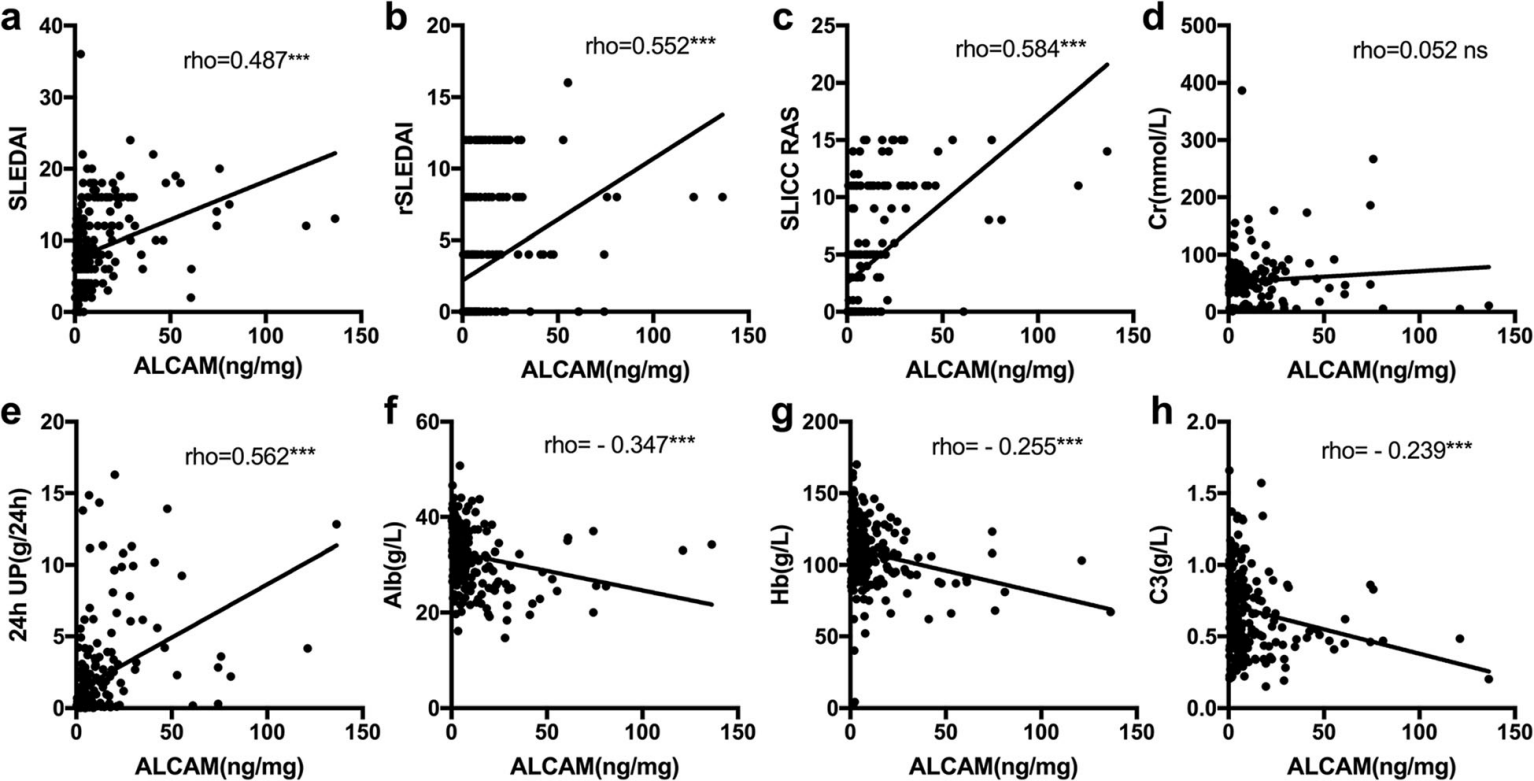
Correlation between urinary ALCAM and **a** SLEDAI, **b** rSLEDAI, **c** SLICC RAS, **d** serum creatinine, **e** 24-h urine protein, **f** serum albumin, **g** hemoglobulin, and **h** complement 3. SLEDAI, Systemic Lupus Erythematosus Disease Activity Index; rSLEDAI, renal SLEDAI; SLICC RAS, SLICC renal activity score; Cr, creatinine; 24-h UP, 24-h urine protein; AIb, serum albumin; Hb, hemoglobin; C3, complement 3

To further assess the relationship between urinary ALCAM and disease activity, we performed a correlation analysis between urinary ALCAM levels and disease activity index including SLEDAI for global disease activity and rSLEDAI and SLICC RAS for renal disease activity. We found a positive correlation between urinary ALCAM with both global disease activity (vs SLEDAI, *r* = 0.487, *p* < 0.001) and renal disease activity (vs rSLEDAI, *r* = 0.552 *p* < 0.001; vs SLICC RAS, *r* = 0.584, *p* < 0.001) (Fig. 2a–c). Besides disease activity, we also explored the correlation between urinary ALCAM and other clinical and laboratory parameters. There was no significant correlation between urinary ALCAM and age, or sex (Supplemental Table 1). No significant correlation was established between urinary ALCAM and any clinical manifestations listed in Table 1 (Supplemental Table 1). However, urinary ALCAM levels correlated positively with 24-h urine protein (*r* = 0.562, *p* < 0.001) and negatively with serum albumin (*r* = − 0.347, *p* < 0.001), hemoglobin level (*r* = − 0.255, *p* < 0.001), and C3 (*r* = − 0.239, *p* < 0.001) (Fig. 2e–g). No significant correlation between urinary ALCAM and serum creatinine (Fig. 2d), serum C4, or anti-dsDNA antibody level was observed.

### Urinary ALCAM as a biomarker of renal pathology and activity index

**Fig. 3:**
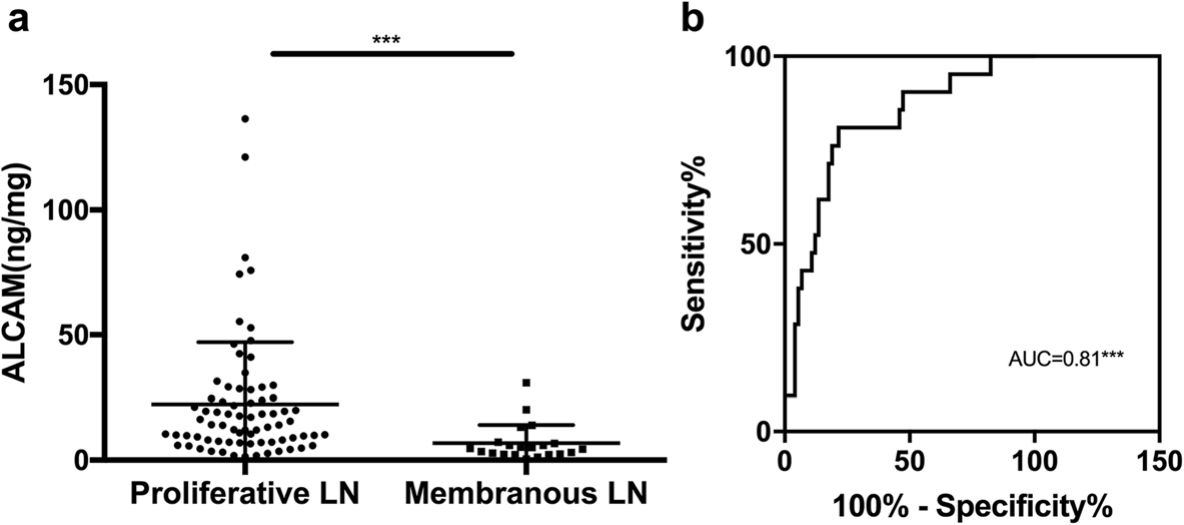
Urinary ALCAM levels were elevated in proliferative LN patients. **a** Group comparison between proliferative (class III/IV ± V) LN patients (14.10 IQR (18.21) ng/mg) and membranous (class V) LN patients (4.70 IQR (4.56) ng/mg). **b** Receiver operating characteristic curve analysis of urinary ALCAM in distinguishing proliferative LN from membranous LN. LN, lupus nephritis; AUC, area under the curve

**Fig. 4:**
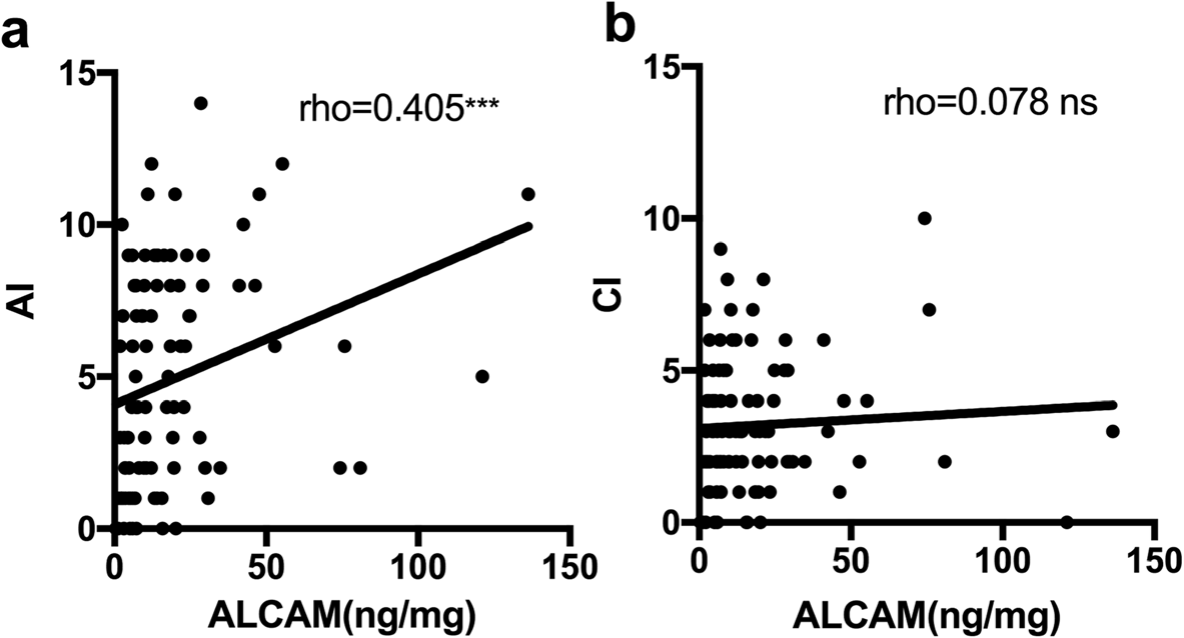
Correlation analysis between urinary ALCAM and **a** AI and **b** CI in renal histopathology. AI, activity index; CI, chronicity index

In the current study, all 96 patients in the active LN group had a concurrent renal biopsy at the time the urine samples were collected. This provided a unique opportunity to assess the performance of urinary ALCAM as a biomarker for assessing renal pathology in LN. Among these patients, there were 1 (1.0%) class II LN, 20 (20.8%) class III/III + V LN, 54 (56.3%) class IV/IV + V LN, and 21 (21.9%) class V. Urinary ALCAM levels were significantly increased in proliferative LN (classes III and IV) (14.10 IQR (18.21) ng/mg) as compared to those in membranous LN (class V) (4.70 IQR (4.56) ng/mg, *p* < 0.001) (Fig. 3a). ROC analysis indicated that urinary ALCAM performed well in discriminating proliferative LN from membranous LN (AUC = 0.81, 95% CI 0.70–0.92, *p* < 0.001) (Fig. 3b), outperforming the traditional markers C3 (AUC = 0.77, 95% CI 0.65–0.89), C4 (AUC = 0.58, 95% CI 0.43–0.72), dsDNA antibody (AUC = 0.58, 95% CI 0.42–0.75), and 24-h urine protein (AUC = 0.59, 95% CI 0.45–0.73) (Table 2). Urinary ALCAM had the highest sensitivity, specificity, PPV, and NPV among these markers (Table 2). More importantly, urinary ALCAM levels correlated positively with AI (*r* = 0.405, *p* < 0.001) but not CI (*r* = 0.08, *p* = 0.448) in renal histopathology, supporting a role for this biomarker in guiding clinical management of LN (Fig. 4a, b).

**Table 2:** Diagnostic performance of urinary ALCAM and conventional biomarkers in discriminating proliferative LN from membranous LN

	Cutoff	AUC	95% CI	SEN (%)	SPE (%)	PPV (%)	NPV (%)
ALCAM (ng/mg)	> 12	0.81	0.70–0.92	78.4	81.0	93.5	51.5
dsDNA (IU/mL)	> 16.5	0.58	0.42–0.75	74.5	50.0	79.5	42.9
C3 (g/L)	< 0.73	0.77	0.65–0.89	75.0	73.7	91.5	43.8
C4 (g/L)	< 0.16	0.58	0.43–0.72	79.7	36.8	82.1	33.3
24 h UP (g/24 h)	> 2.8	0.59	0.45–0.73	59.7	61.9	84.3	31.0

*AUC* area under the curve, *CI* confidence interval, *SEN* sensitivity, *SPE* specificity, *PPV* positive predictive value, *NPV* negative predictive value, *24-h UP* 24-h urine protein

## Discussion

In this cross-sectional study, we performed a validation of urinary ALCAM as a potential biomarker for lupus nephritis in a Chinese lupus cohort. We were able to demonstrate that the levels of urinary ALCAM were elevated in active LN patients as compared to active SLE without renal involvement, inactive LN, inactive SLE patients without renal involvement, and healthy controls. It correlated with global disease activity as well as renal disease activity. In the subgroup of biopsy-concurrent LN patients, urinary ALCAM was significantly increased in patients with proliferative LN compared to those with membranous LN and outperformed conventional biomarkers (anti-dsDNA antibody, C3, C4, 24-h urine protein) in discriminating the two different types of LN. Furthermore, urinary ALCAM levels correlated positively with activity index (AI) in renal histopathology. Taken together, our results suggest urinary ALCAM is a promising biomarker for predicting renal histopathological changes in LN.

ALCAM is a cell surface glycoprotein which is shed into body fluid by proteolytic cleavage [7, 8]. The major function of ALCAM includes mediating immune cell adhesion and migration, promoting T cell activation and proliferation [10, 11, 17]. ALCAM has been increasingly used as a biomarker for the diagnosis, treatment response, and survival prediction in various cancers [18–20]. Recently, a urinary proteome profiling study identified several increased urinary proteins including ALCAM in type 1 diabetes patients as compared to their healthy siblings, alluding to its potential role in the pathogenesis of intermittent hyperglycemia and inflammation [21]. In type 2 diabetic nephropathy, serum ALCAM level and ALCAM expression in the glomeruli were significantly increased, suggesting ALCAM as a potential mediator in the late complications of diabetes in the kidney [22]. The role of ALCAM in lupus nephritis has been only reported in a microarray study of MRL/lpr LN mice, which revealed increased ALCAM expression in the glomeruli [12]. Taken together with our recent aptamer-based proteomic screening study [6], urinary ALCAM warrants further validation as a biomarker in LN.

In the current study, we observed significantly increased urinary ALCAM in active LN patients compared to active SLE patients without renal involvement, indicating an exclusive role for ALCAM in renal involvement of SLE. Correlation analysis revealed that urinary ALCAM levels correlated significantly with both global disease activity and renal disease activity. When we did a subgroup correlation analysis, in active SLE patients without renal involvement, no positive correlation between urinary ALCAM and global disease activity was seen (data not shown), while in the active LN group, there was a significant positive correlation, indicating that the positive correlation between urinary ALCAM and SLEDAI is likely due to the renal components of the SLEDAI score. The positive correlation between urinary ALCAM and 24-h urine protein in the current study also suggests that ALCAM could be an indicator of renal damage in LN. Moreover, urinary ALCAM was not correlated with serum creatinine level in this study, indicating that the change in urinary ALCAM was not likely to be impacted by renal function. Indeed, the increased urinary ALCAM levels in active LN patients may reflect an increased expression of ALCAM in the kidney, which is in accordance with the microarray study in LN mice [12]. The expression of ALCAM in LN patients’ kidneys and its relationship with disease activity is currently under active investigation.

An important finding was that urinary ALCAM was elevated in proliferative LN as compared to membranous LN, rendering ALCAM a potential biomarker to differentiate proliferative LN from membranous LN. This is of clinical importance since the LN guidelines recommend management of LN based on the histopathological classification of LN [23, 24]. In these guidelines, patients with proliferative LN (class III/IV ± V) and membranous LN (class V) are recommended with different induction therapy. The ability of urinary ALCAM to differentiate the two groups of LN patients might be useful in the situation where renal biopsy is contraindicated or when patients are reluctant to do a biopsy. Compared to conventional markers, such as 24-h urine protein, AUC for ALCAM exceeded AUC for proteinuria in distinguishing class III/IV from V. Conventional markers have been examined in various studies as biomarkers of renal disease activity [4, 25, 26]. Here, we show that urinary ALCAM outperforms conventional markers as a biomarker for differentiating class III/IV ± V LN from pure class V LN. More interestingly, urinary ALCAM also correlated with renal pathology activity index but not the chronicity index, which is concordant with the observed correlation with clinical renal disease activity. Activity index in renal histopathology has been documented to be a predictor of long-term renal outcome in different cohorts [27–29]. The correlation between urinary ALCAM and activity index suggests a prediction potential of urinary ALCAM for long-term renal outcome, which warrants further investigation in a longitudinal cohort.

Previous studies have found elevated VCAM-1 and ICAM-1 to be potential biomarkers for active LN [30–38] and elevated VCAM-1 as a predictor for renal pathology activity index in LN [35, 36]. Like ALCAM, both VCAM-1 and ICAM-1 are immunoglobulin superfamily cell adhesion molecules (Ig SF CAMs). Although urinary VCAM-1 and ICAM-1 have been widely validated as biomarkers for LN, the role of urinary ALCAM as a biomarker for LN has been validated only recently by Parodis et al. in a European cohort [38]. In their study, urinary ALCAM was associated with LN history in SLE patients and higher urinary VCAM-1 and ALCAM predicted long-term renal function deterioration. Although the characteristics of patients in their study were considerably different from the current study, both studies confirmed the association of ALCAM and renal affliction. Taken together, one could potentially develop a panel of the abovementioned cell adhesion molecules to predict renal disease activity in LN in future studies. Although previous studies on VCAM-1 and ICAM-1 as biomarkers of LN had consistent results among populations with different ethnicity, they have not assessed the ability of the two adhesion molecules to differentiated proliferative LN from membranous LN. We would include the three cell adhesion molecules in our prospective LN biomarker validation study. Moreover, given that these three Ig SF CAM members have been documented to be potential biomarkers in active LN, they suggest a potential pathogenic role of cell adhesion molecules in LN. Given the important role of ALCAM in T cell activation and in mediating cell adhesion to the endothelium [10], it is reasonable to hypothesize that ALCAM plays a pivotal role in the development of LN, which is worthy of mechanistic studies in the future.

Given the fact that the expression of ALCAM was also increased in type 2 diabetic nephropathy patients’ glomeruli, ALCAM might not be specific to renal disease in SLE. The increased urinary ALCAM might reflect series processes of inflammation at the kidney level. One of the limitations of the current study is the lack of chronic kidney disease patients as disease controls. However, our goal in this study was not to validate the diagnostic role of urinary ALCAM in differentiating LN from other chronic kidney diseases. Instead, we focused on the role of urinary ALCAM in gauging renal pathology in LN patients. Given the limited number of inactive LN patients in this study, further validation studies are needed before urinary ALCAM can be considered for clinical practice.

## Conclusion

In summary, our observations indicate that urinary ALCAM is a potential non-invasive biomarker of renal histopathology in LN patients. Urinary ALCAM showed ability to discriminate class III/IV from V lupus nephritis. It outperformed conventional markers (anti-dsDNA antibody, C3, C4, 24-h urine protein) in distinguishing class III/IV ± V from pure class V LN. Urinary ALCAM is reflective of renal pathology activity index in lupus nephritis.

## Supplementary information

**Additional file 1 : Supplemental Table 1**. Correlation analysis of ALCAM and patients’ characteristics.

## Data Availability

The datasets generated and/or analyzed during the current study available from the corresponding author on reasonable request.

## Abbreviations

LN: Lupus nephritis
SLE: Systemic lupus erythematosus
dsDNA: Anti-double-stranded DNA
ALCAM or CD166: Activated leukocyte cell adhesion molecule
ACR: American College of Rheumatology
SLEDAI: Systemic Lupus Erythematosus Disease Activity Index
SLICC RAS: SLICC renal activity score
ISN/RPS: International Society of Nephrology/Renal Pathological Society
aLN: Active LN group
aNR: Active SLE without renal involvement group
iLN: Inactive LN patients
iNR: Inactive SLE patients without renal involvement
C3: Complement component 3
C4: Complement component 4
IQR: Interquartile range
SD: Standard deviation
ANOVA: One-way analysis of variance
ROC: Receiver operating characteristic
PPV: Positive predictive value
NPV: Negative predictive value
AUC: Area under the curve
AI: Activity index
CI: Chronicity index
