# Supplementary material for: Urinary activated leukocyte cell adhesion molecule as a novel biomarker of lupus nephritis histology
Source: Arthritis Res Ther. 2020 May 27;22:122. doi: 10.1186/s13075-020-02209-9 (PMC7251704; doi:10.1186/s13075-020-02209-9)
Supplement: Supplementary file 1 — Additional file 1 : Supplemental Table 1. Correlation analysis of ALCAM and patients’ characteristics. [file 13075_2020_2209_MOESM1_ESM.docx]

Supplemental Table 1. Correlation analysis of ALCAM and patients’ characteristics

|  | Spearman's Rho | | P value | |
| --- | --- | --- | --- | --- |
| Sex | 0.061 | 0.331 | |  |
| Age | 0.088 | 0.159 | |  |
| Disease Course | 0.04 | 0.557 | |  |
| Fever | -0.101 | 0.129 | |  |
| Lymphadenopathy | 0.05 | 0.457 | |  |
| Malar rash | -0.057 | 0.394 | |  |
| Mucosal ulceration | -0.008 | 0.905 | |  |
| Alopecia | 0.063 | 0.346 | |  |
| Vasculitis | 0.111 | 0.094 | |  |
| Raynoud's phenomenon | 0.005 | 0.935 | |  |
| NPSLE | -0.004 | 0.956 | |  |
| Myositis | -0.035 | 0.601 | |  |
| Arthralgia/arthritis | -0.031 | 0.637 | |  |
| Endocarditis | 0.071 | 0.285 | |  |
| PAH | -0.054 | 0.418 | |  |
| ILD | -0.012 | 0.851 | |  |
| Pleural efusion | 0.035 | 0.602 | |  |
| Hemolytic anemia | -0.094 | 0.155 | |  |
| Leukocytopenia | 0.039 | 0.555 | |  |
| Thrombocytopenia | -0.018 | 0.783 | |  |
| Gastrointestinal vasculitis | -0.119 | 0.072 | |  |

NPSLE: Neuropsychiatric systemic lupus erythematosus; PAH: pulmonary arterial hypertension; ILD: interstitial lung disease;
